# The impact of dog phobia severity on views regarding stray dog management in Türkiye

**DOI:** 10.3389/fvets.2025.1548580

**Published:** 2025-05-07

**Authors:** Vasfiye Demir Pervane, Betül Uyar, Pakize Gamze Erten Bucaktepe

**Affiliations:** ^1^Family Medicine Department, Faculty of Medicine, Dicle University, Diyarbakir, Türkiye; ^2^Psychiatry Department, Faculty of Medicine, Dicle University, Diyarbakir, Türkiye

**Keywords:** dog phobia, stray dogs, free-roaming dogs, dog management, shelter

## Abstract

**Background and objectives:**

The aim of this study was to evaluate the opinions of individuals regarding the management of stray dogs and its relationship with dog phobia.

**Materials and methods:**

The study was a cross-sectional analytical study, and data were collected online across Türkiye. Of 1,359 participants, 63.1% of the participants were female and 89.6% were university graduates. 69.5% of participants had any level of dog phobia. Younger individuals, those without pets or experience with stray animals, and those with a history of dog attacks showed higher phobia rates (all *p* < 0.001). Fear of stray dogs, being attacked, and contracting rabies were common concerns, correlating with higher phobia levels (*p* < 0.001). The rates of supporting vaccinating and neutering dogs were 92.3 and 49.8%, respectively. Of the respondents, 53.3% agreed with keeping neutered dogs in shelters. It has been found that people who do not own dogs [OR: 1.779 (95% CI: 1.005–3.150), *p* = 0.048] and cats [OR: 1.931 (95% CI: 1.044–3.572), *p* = 0.036]; who had never provided care for stray animals [OR: 2.034 (95% CI: 1.467–2.821) *p* < 0.001] and those with a personal or family history of having been attacked by a dog than in those [OR: 2.101 (95% CI: 1.631–2.706) *p* < 0.001] are approximately twice as likely to have a phobia. Participants with dog phobia were 65.5% less likely to agree that dogs that have been vaccinated and neutered should be left on the streets again [OR: 0.345 (95% CI: 0.270–0.441) *p* < 0.001], and those with dog phobia were 2.6 times more likely to state that dogs that have been vaccinated and neutered should be kept in shelters and given the necessary care [OR: 2.649 (95% CI: 2.078–3.377) *p* < 0.001].

**Conclusion:**

Dog phobia is prevalent, and stray dog management significantly influences public fear levels and perceptions. Understanding the relationship between dog phobia and attitudes toward stray dog management can help inform policies that address both public concerns and animal welfare. More representative studies are needed to better reflect the general population.

## 1 Introduction

The current global situation regarding the stray animal population is overwhelming, and it has elicited varied responses from both communities and individuals. Evidence suggests that dog populations total 900 million, of which 83% are free-roaming strays. Public opinion on the management of stray animals reflects diverse perspectives, ranging from public safety and health concerns to ethical considerations of animal rights and welfare. Stray dog populations have been linked to potential risks such as zoonotic diseases, traffic accidents, and, in some cases, aggressive encounters with humans (1).

The complexity of managing stray animals lies in balancing public safety with ethical responsibilities, and it is often influenced by personal experiences and psychological factors. One such factor that may influence these opinions is dog phobia, a specific phobia characterized by an intense and irrational fear of dogs (2). Dog phobia is categorized within specific phobias according to the Diagnostic and Statistical Manual of Mental Disorders, Fifth Edition (DSM-5), and it can significantly impair the lives of those affected. This phobia can lead to avoidance behaviors, excessive anxiety, and even panic attacks when encountering or thinking about dogs (3).

Previous studies have examined various factors influencing public attitudes toward stray animals and their management (4, 5). A study by Serpell explored the role of cultural, social, and psychological factors in shaping attitudes toward pets and stray animals, finding that negative experiences with animals often contribute to a preference for more restrictive control measures (5). These findings suggest that specific phobia severity could play a role in shaping individual attitudes toward animal management strategies, including stray dogs. People with a high phobia severity may advocate strict precautions around animals, while those with less severe symptoms may support more neutral control measures.

Research on attitudes toward stray dogs has revealed that concerns about public health and safety often drive support for stringent management measures, particularly in areas with high stray dog populations (6). However, studies have also shown that these views vary based on demographic factors, including age, gender, and socioeconomic status, as well as personal experiences with animals. For example, people with positive past experiences with animals are more likely to support humane management strategies, such as adoption and sterilization programs (7). Conversely, individuals who report negative or fearful experiences, particularly with dogs, may favor euthanasia or other restrictive measures. In this context, dog phobia could be an important determinant, as those with a higher phobia severity may perceive stray dogs as a direct threat to their safety.

There is a gap in the literature regarding studies examining the relationship between dog phobia severity and individuals’ views on stray animal management. By examining this relationship, the aim of this study was to determine whether psychological factors, specifically specific phobia severity, influence public attitudes toward animal management policies such as euthanasia, neutering, or adoption. Understanding the impact of dog phobia severity on views on stray dog management has broader implications for public policy. Effective stray dog management requires balancing safety concerns with ethical considerations and the humane treatment of animals. Policymakers can benefit from insights into how psychological factors, such as specific phobia severity, shape public opinion. These insights can then help in the development of strategies to address public concerns while also promoting humane and sustainable management practices for stray animals.

## 2 Materials and methods

This research was designed as a cross-sectional, analytical study, and the Strengthening the Reporting of Observational Studies in Epidemiology (STROBE) guidelines were followed during preparation (8). Approval for the study was granted by the Non-Interventional Research Ethics Committee of Dicle University (decision no: 267, date: 25 September 2024).

For data collection, an online questionnaire was prepared and then delivered via online platforms to individuals aged 18–65 years throughout the 7 geographic regions of Türkiye. The number of subjects to be included was calculated in order to estimate the prevalence of dog phobia with a 90% confidence and a precision of 2.5%. This yields a minimum sample size of 1,082 people. A slightly larger sample of 1,359 people was achieved. The participant were distributed proportional to the population of the seven geographical regions considered. The snowball sampling method was used in data collection.

After receiving approval from the ethics committee, data were collected between 1 October 2024 and 24 October 2024. The questionnaire, prepared on Google Forms, was delivered online. The study was conducted in compliance with the “Informed Consent, Confidentiality and Privacy Protection, and Respect for Autonomy” ethical principles and the Helsinki Declaration.

The inclusion criteria for the study were determined as age in the range of 18–65 years and voluntary participation. The data form and scale were prepared using Google Forms, including information about the study and a box to be marked indicating informed consent for participation before the completion of the questionnaire. The completed questionnaires were transferred to Microsoft Excel, and, after the data were checked, they were uploaded to a statistical program for analysis.

### 2.1 Variables

A total of 19 parametres were evaluated, including sociodemographic characteristics (age, gender, education level, marital status, number of children, place of residence, and geographic region) and pet ownership characteristics (the presence of a pet in the home, what kind of pet, duration of pet ownership, experience of caring for stray animals, history of being attacked by a dog, level of fear of dogs and opinions on stray dog management). Each province in Türkiye has a licence plate code identified by a number. In order to categorize the geographical regions in which the participants participated, the plate code of the province they lived in was asked and then categorized according to geographical regions. The level of fear of dogs was evaluated on a 5-point Likert-type scale ranging from “I am not at all afraid” to “I am very afraid,” and the questions related to the management of stray animals had 3-point Likert-type responses of “Yes, No, Undecided.” The level of fear was then reclassified in 3 groups as “I am not afraid,” “I am neither afraid nor not afraid,” and “I am afraid,” and analyses were made accordingly. The questions were all prepared by the researchers (see Supplementary material).

Dog phobia was examined using the DSM-5 Severity Measure for Specific Phobia Scale-Adult version, which was developed by the American Psychiatric Association (9). Öztekin et al. (10) conducted validity and reliability studies of the Turkish version of the scale, which is a 10-item scale evaluating the severity of a specific phobia in individuals aged ≥18 years with a 0.79 cronbach alpha value. For each item, subjects are requested to grade the severity of specific phobia symptoms experienced in the last 7 days. In the scoring and interpretation of the scale, each item is graded from 5 points (0 = never, 1 = sometimes, 2 = half the week, 3 = most of the week, and 4 = all week). The total score ranges from 0 to 40 points, with higher points indicating more severe specific phobia symptoms. The raw score is obtained as the total of the raw points obtained from the 10 items, and then the average total score is calculated by dividing the total raw points by the number of items in the scale. The average total score converts the overall score to a 5-point scale, allowing the clinician to evaluate the specific phobia severity of an individual as none (0), mild (1), moderate (2), severe (3), and very severe (4). Studies in the field of DSM-5 have found the average total score to be reliable, easy to use, and helpful for clinicians. The participants were asked to fill out the scale considering the dog as a specific phobia. The data form was prepared in Turkish, and no alternative was offered to the participants to be filled in another language. The presence of dog phobia was categorized into two groups: ‘no dog phobia’ and ‘dog phobia present’ (including any level of phobia from mild to extreme). Comparisons were then conducted based on these categories.

### 2.2 Statistical analysis

The data obtained in this study were analyzed statistically using SPSS (Statistical Package for Social Sciences) for Windows vn. 27.0 software (IBM Corp., Armonk, NY, USA). Numerical variables are presented as mean ± standard deviation (SD) values, and categorical variables are presented as number (*n*) and percentage (%). The normality of the data was assessed using the Kolmogorov–Smirnov test, histograms, and skewness-kurtosis values, revealing that the data did not follow a normal distribution. The Chi-square test (χ2) was used to compare categorical data, and a Spearman rho correlation analysis was applied to determine relationships between continuous data. When significance was determined in more than two groups of categorical data, a Bonferroni- corrected post -hoc analysis was performed to determine from which category the significance originated. To determine predictors of dog phobia, variables determined at a significance level of *p* < 0.25 in a univariate analysis were included in a multivariate logistic regression analysis (Enter method). In the current study, the internal consistency coefficient (Cronbach alpha value) was calculated and the results found to be 0.942 for this sample regarding the specific phobia scale. The hypotheses were two-way, and the level of statistical significance was accepted as *p* < 0.05 in a 95% confidence interval.

## 3 Results

A total of 1,359 individuals participated in this study, comprising 858 (63.1%) females and 501 (36.9%) males, with a mean age of 35.9 ± 10.4 years (range of 18–65 years). The level of education was university or above for 89.6% of the sample, and 55.3% were married (Table 1). Pet ownership was reported by 485 (35.7%) participants, with a mean pet ownership duration of 5.9 ± 6.2 years. It was stated by 76.4% of the participants that they had previously provided care for stray animals (cat or dog), and 46.1% had a history of themselves or a family member having been attacked by a dog.

**Table 1:** Descriptive characteristics of the study participants.

Sociodemographic characteristics		*n*	%
Gender	Female	858	63.1
	Male	501	36.9
Age (years)	18–29	443	32.6
	30–39	427	31.4
	40–49	319	23.5
	50–65	170	12.5
Education level	High school and below	141	10.4
	University and above	1,218	89.6
Marital status	Married	751	55.3
	Not married	608	44.7
Geographic region	Marmara	169	12.4
	Aegean	84	6.2
	Mediterranean	83	6.1
	South-east	558	41.1
	Central Anatolia	326	24.0
	East Anatolia	57	4.2
	Black Sea	82	6.0
Children	None	679	50.0
	Yes	680	50.0
Owning a pet	No	874	64.3
	Yes	485	35.7
Providing care for stray animals (cat/dog)	No	321	23.6
	Yes	1,038	76.4
A history of themself or a family member having been attacked by a dog	No	733	53.9
	Yes	626	46.1

The points obtained from the Dog Phobia Scale were totaled and categorized according to the presence of a dog phobia and severity (Table 2). While 30.5% of the participants had no dog phobia, 69.5% had a dog phobia at any level (from mild to very severe). Additionally, 5.8% of the participants were found to have a severe or very severe dog phobia.

**Table 2:** Mean scores of dog phobia severity.

Level of dog phobia	*n*/Mean ± SD	%/Min–Max
None (0 points)	414	30.5
Mild (1–10 points)	721	53.1
Moderate (12–20 points)	145	10.7
Severe (21–30 points)	59	4.3
Very severe (31–40 points)	20	1.5
Phobia present—total	945	69.5
Phobia scale points	5.7 ± 7.4	0–40

The dog phobia status of the participants was compared with sociodemographic data and descriptive characteristics related to pet ownership and care (Table 3). Dog phobia was determined to be present at a statistically significantly higher rate in the participants aged 18–29 years (*p* < 0.001), those who did not own any pet (*p* < 0.001), those who did not own a cat or dog (*p* < 0.001), those who had not provided care for stray animals (*p* < 0.001), and those with a personal or family history of being attacked by a dog (*p* < 0.001). While there was no relationship between gender and education level and the presence of dog phobia, a significant relationship was found between the distribution of the participants according to geographical regions and the presence of dog phobia (*p* = 0.009). The highest dog phobia rate was found in the Eastern Anatolia region (87.7%).

**Table 3:** Comparisons of dog phobia according to descriptive characteristics.

Characteristics		Dog Phobia		p*
		Absent (%)	Present (%)	
Gender	Female	254 (29.6)	604 (70.4)	0.367
	Male	160 (31.9)	341 (68.1)	
Age (years)	18–29	100 (22.6)	343 (77.4)	<0.001
	30–39	121 (28.3)	306 (71.7)	
	40–49	123 (38.6)	196 (61.4)	
	50–65	70 (41.2)	100 (58.8)	
Education level	High school and below	50 (35.5)	91 (64.5)	0.173
	University and above	364 (29.9)	854 (70.1)	
Marital status	Married	237 (31.6)	514 (68.4)	0.330
	Not married	177 (29.1)	431 (70.9)	
Geographic region	Marmara	52 (30.8)	117 (69.2)	0.009
	Aegean	35 (41.7)	49 (58.3)	
	Mediterranean	30 (36.1)	53 (63.9)	
	South-east	163 (29.2)	395 (70.8)	
	Central Anatolia	106 (32.5)	220 (67.5)	
	East Anatolia	7 (12.3)	50 (87.7)	
	Black Sea	21 (25.6)	61 (74.4)	
Children	None	198 (29.2)	481 (70.8)	0.297
	Yes	216 (31.8)	464 (68.2)	
Owning a pet	No	202 (23.1)	672 (76.9)	<0.001
	Yes	212 (43.7)	273 (56.3)	
Owning a cat	No	250 (24.9)	756 (75.1)	<0.001
	Yes	164 (46.5)	189 (53.5)	
Owning a dog	No	371 (29.2)	899 (70.8)	<0.001
	Yes	43 (48.3)	46 (51.7)	
Owning another animal	No	371 (29.8)	873 (70.2)	0.092
	Yes	43 (37.4)	72 (62.6)	
Providing care for stray animals (cat/dog)	No	59 (18.4)	262 (81.6)	<0.001
	Yes	355 (34.2)	683 (65.8)	
A history of themself or a family member having been attacked by a dog	No	269 (36.7)	464 (63.3)	<0.001
	Yes	145 (23.2)	481 (76.8)	

^*^Chi-square analysis.

When the opinions of the participants about the management of stray dogs were evaluated, it was found that 92.3% stated that all dogs should be vaccinated, 49.8% stated that all dogs should be neutered, 38.5% stated that all neutered dogs should be returned to the streets, and 53.3% stated that all vaccinated and neutered dogs should be kept in shelters and given the necessary care (Table 4). Of the total participants, 67.8% stated that they were not afraid of owned dogs, 50.2% were afraid of stray dogs, 63.0% were afraid of being attacked by a stray dog, and 67.0% were afraid of the transmission of rabies from a stray dog. The views of the participants about the management of stray animals and dog fear status were also compared according to the presence of dog phobia (Table 4). Statistically significant differences were observed between the responses to all views except the statement that all dogs should be vaccinated and the presence of dog phobia. It was observed that those with dog phobia were more likely to be undecided about whether dogs should be neutered (31.6% versus 25.8%). Those with a dog phobia were less likely to agree that vaccinated and neutered dogs should be released back onto the streets (30.7% versus 56.3%) and were more likely to believe that such dogs should be kept in shelters and provided with the necessary care (60.7% versus 36.2%). In the evaluation of fear, it was observed that those with dog phobia reported higher rates of fear of owned pet dogs (26.5% versus 3.1%), stray dogs (65.7% versus 14.7%), the possibility of being attacked by a stray dog (78.3% versus 28.3%), the possibility of contracting rabies from a stray dog (79.4% versus 38.6%), compared to those without dog phobia.

**Table 4:** Distribution of participants’ views on the management of stray animals and comparison with dog phobia status.

	All participants views *n* (%)	Dog phobia		p*
		Absent *n* (%)	Present *n* (%)	
All dogs should be vaccinated				
No	33 (2.4)	10 (2.4)	23 (2.4)	0.295
Undecided	72 (5.3)	16 (3.9)	56 (5.9)	
Yes	1,254 (92.3)	388 (93.7)	866 (91.6)	
All dogs should be neutered				
No	276 (20.3)	79 (19.1)	197 (20.8)	0.031
Undecided	406 (29.9)	107 (25.8)	299 (31.6)	
Yes	677 (49.8)	228 (55.1)	449 (47.5)	
Dogs that have been vaccinated and neutered should be left on the streets again				
No	407 (29.9)	80 (19.3)	327 (34.6)	<0.001
Undecided	429 (31.6)	101 (24.4)	328 (34.7)	
Yes	523 (38.5)	233 (56.3)	290 (30.7)	
Dogs that have been vaccinated and neutered should be kept in shelters and given the necessary care				
No	298 (21.9)	151 (36.5)	147 (15.6)	<0.001
Undecided	337 (24.8)	113 (27.3)	224 (23.7)	
Yes	724 (53.3)	150 (36.2)	574 (60.7)	
When encountering owned pet dogs				
I am not afraid	921 (67.8)	381 (92.0)	540 (57.1)	<0.001
Neither afraid nor not afraid	175 (12.9)	20 (4.8)	155 (16.4)	
I am afraid	263 (19.4)	13 (3.1)	250 (26.5)	
When encountering stray dogs				
I am not afraid	489 (36.0)	285 (68.8)	204 (21.6)	<0.001
Neither afraid nor not afraid	188 (13.8)	68 (16.4)	120 (12.7)	
I am afraid	682 (50.2)	61 (14.7)	621 (65.7)	
The possibility of being attacked by a stray dog				
I am not afraid	353 (26.0)	208 (50.2)	145 (15.3)	<0.001
Neither afraid nor not afraid	149 (11.0)	89 (21.5)	60 (6.3)	
I am afraid	857 (63.0)	117 (28.3)	740 (78.3)	
The possibility of contracting rabies from a stray dog				
I am not afraid	317 (23.3)	184 (44.4)	133 (14.1)	<0.001
Neither afraid nor not afraid	132 (9.7)	70 (16.9)	62 (6.6)	
I am afraid	910 (67.0)	160 (38.6)	750 (79.4)	

***Chi-square test.

It was observed that as the severity level of dog phobia of the participants varied, the rate of responses to their opinions on the management of dogs also varied. The difference in the opinion regarding dog vaccination did not vary significantly in relationship with levels of dog phobia (*p* = 0.718). The proportion of participants stating that dogs should be neutered increased with phobia severity ranging from 44.4% among those with mild dog phobia to 70.0% among those with extreme dog phobia (*p* = 0.012). As the severity of dog phobia increased, the proportion of participants supporting the return of sterilized dogs to the streets decreased, from 35.6% among individuals with mild dog phobia to 10% among those with extreme dog phobia (*p* < 0.001). In contrast, the proportion of participants favoring the sheltering and care of vaccinated and neutered dogs increased with phobia severity, ranging from 55.2% among those with mild dog phobia to 90.0% among those with extreme dog phobia (*p* < 0.001) (Table 5).

**Table 5:** Opinions about the management of stray animals according to the severity of dog phobia (*n* = 945).

		Dog phobia severity categories				p*
		Mild	Moderate	Severe	Very severe	
		*n* (%)	*n* (%)	*n* (%)	*n* (%)	
All dogs should be vaccinated	No	17 (2.4)	4 (2.8)	2 (3.4)	0 (0)	0.718
	Undecided	44 (6.1)	5 (3.4)	5 (8.5)	2 (10.0)	
	Yes	660 (91.5)	136 (93.8)	52 (88.1)	18 (90.0)	
All dogs should be neutered	No	166 (23.0)	23 (15.9)	6 (10.2)	2 (10.0)	0.012
	Undecided	235 (32.6)	41 (28.3)	19 (32.2)	4 (20.0)	
	Yes	320 (44.4)	81 (55.9)	34 (57.6)	14 (70.0)	
Dogs that have been vaccinated and neutered should be left on the streets again	No	214 (29.7)	65 (44.8)	34 (57.6)	14 (70.0)	<0.001
	Undecided	250 (34.7)	57 (39.3)	17 (28.8)	4 (20.0)	
	Yes	257 (35.6)	23 (15.9)	8 (13.6)	2 (10.0)	
Dogs that have been vaccinated and neutered should be kept in shelters and given the necessary care	No	134 (18.6)	9 (6.2)	3 (5.1)	1 (5.0)	<0.001
	Undecided	189 (26.2)	26 (17.9)	8 (13.6)	1 (5.0)	
	Yes	398 (55.2)	110 (75.9)	48 (81.4)	18 (90.0)	

^*^Chi-square analysis.

In the correlation analysis, it was found that, as the levels of fear of owned dogs, stray dogs, being attacked by a stray dog, and contracting rabies from a stray dog increased, there was an increase in the Dog Phobia Scale points (Table 6). A negative correlation was determined between age, the duration of pet ownership, and the Dog Phobia Scale points.

**Table 6:** Correlations.

	1	2	3	4	5	6	7
Age (1)							
Number of children (2)	0.655 **						
Duration of pet ownership (3)	0.176 **	−0.003					
Phobia scale (dog) points (4)	−0.163 **	0.023	−0.189 **				
Fear of owned pet dogs (5)	−0.052	0.079 **	−0.159 **	0.482 **			
Fear of stray dogs (6)	−0.119	0.032	−0.197 **	0.611 **	0.655 **		
Fear of being attacked by a stray dog (7)	−0.169	−0.005	−0.235 **	0.585**	0.580 **	0.854 **	
Fear of contracting rabies from a stray dog (8)	−0.175 **	−0.043	−0.248 **	0.485 **	0.440 **	0.680 **	0.785 **

^**^p < 0.001 level of statistical significance.

According to the logistic regression analysis, the dog phobia was approximately 60% less frequent in the 50–65 years age group than in the 18–29 years age group [OR: 0.419 (95% CI: 0.281–0.623) *p* < 0.001]. It has been found that people who do not own of dogs [OR: 1.779 (95% CI: 1.005–3.150) *p* = 0.048] and cats [OR: 1.931 (95% CI: 1.044–3.572) *p* = 0.036] are approximately 2 times more likely to be phobic. A 2-fold frequent dog phobia was determined in those who had never provided care for stray animals than in those who had [OR: 2.034 (95% CI: 1.467–2.821) *p* < 0.001] and in those with a personal or family history of having been attacked by a dog than in those who did not have such a history [OR: 2.101 (95% CI: 1.631–2.706) *p* < 0.001] (Table 7). Taking dog phobia as an independent variable, we evaluated the opinions on the management of stray animals and the effect of the presence of dog phobia on these opinions (Table 8). It was observed that those with dog phobia were 65.5% less likely to agree that dogs that have been vaccinated and neutered should be left on the streets again [OR: 0.345 (95% CI: 0.270–0.441) *p* < 0.001], and those with dog phobia were 2.6 times more likely to state that dogs that have been vaccinated and neutered should be kept in shelters and given the necessary care [OR: 2.649 (95% CI: 2.078–3.377) *p* < 0.001]. Although the rate of stating that dogs that have been vaccinated and neutered should be kept in shelters and given the necessary care was 2.6 times higher in those with dog phobia, the Hosmer-Lemeshow test result was below 0.05 and the model fit was not found to be good.

**Table 7:** Multivariate logistic regression analysis of the predictive factors of dog phobia.

Variables		Dog phobia present	
		OR (95% CI)	p*
Age (years)	18–29	Reference	
	30–39	0.651 (0.472–0.898)	0.009
	40–49	0.435 (0.311–0.608)	<0.001
	50–65	0.419 (0.281–0.623)	<0.001
Education level	High school and below	Reference	
	University and above	1.250 (0.840–1.860)	0.272
Pet ownership	Yes	Reference	
	No	1.214 (0.626–2.357)	0.566
Ownership of a dog	Yes	Reference	
	No	1.779 (1.005–3.150)	0.048
Ownership of a cat	Yes	Reference	
	No	1.931 (1.044–3.572)	0.036
Ownership of another animal	Yes	Reference	
	No	1.240 (0.678–2.266)	0.485
Caring for stray animals (cat/dog)	Yes	Reference	
	No	2.034 (1.467–2.821)	<0.001
A history of themself or a family member having been attacked by a dog	No	Reference	
	Yes	2.101 (1.631–2.706)	<0.001
Model [χ^2^(10); *p*]	149.960; *p* < 0.001		
Hosmer–Lemeshow test, *p*	0.630		
Nagelkerke R^2^ (%)	14.8		
Classified cases (%)	71.6		

^*^Multivariate logistic regression analysis performed. CI, confidence interval; OR, odds ratio.

**Table 8:** Multivariate logistic regression analysis of views on stray animal management and presence of dog phobia.

		All dogs should be neutered	
		OR (95% CI)	p*
Dog phobia	Absent	Reference	
	Present	0.790 (0.624–1.001)	0.051
	Model [χ^2^(16); *p*]	19.762;<0.001	
	Hosmer–Lemeshow test, *p*	0.354	
	Nagelkerke R^2^ (%)	19.0	
	Classified cases (%)	54.5	

		Dogs that have been vaccinated and neutered should be left on the streets again	
		OR (95% CI)	p*
Dog phobia	Absent	Reference	
	Present	0.345 (0.270–0.441)	<0.001
	Model [χ^2^(16); *p*]	104.015;<0.001	
	Hosmer–Lemeshow test, *p*	0.181	
	Nagelkerke R^2^ (%)	10.0	
	Classified cases (%)	66.7	

		Dogs that have been vaccinated and neutered should be kept in shelters and given the necessary care	
		OR (95% CI)	p*
Dog phobia	Absent	Reference	
	Present	2.649 (2.078–3.377)	<0.001
	Model [χ^2^(16); *p*]	83.031;<0.001	
	Hosmer–Lemeshow test, *p*	0.043	
	Nagelkerke R^2^ (%)	7.9	
	Classified cases (%)	61.6	

^*^Multivariate logistic regression analysis performed. CI, confidence interval; OR, odds ratio. Analysis were adjusted for age and gender.

## 4 Discussion

The bill to amend the Animal Protection Law in Türkiye came into force on 2 August 2024, and these changes allow local authorities to “euthanise” animals taken into shelters who have been designated as aggressive, have an infectious or untreatable disease, or have been prohibited from being owned. In addition, abandoning the “catch–neuter–release” method in the existing law, a move has been made to the “catch–neuter–hold–own” method (11). The new regulation has led to public controversy, and volunteers who care for stray animals and are interested in their rights have stated that the solution primarily offers effective neutering and public education, and the existing law should be reviewed (12). With this study, we wanted to examine the study participants in terms of their opinions about stray dog management and their level of dog phobia, and we wanted to reveal the relationship between the two, considering that the biopsychological aspect may play a role in the decisions and attitudes of individuals in social events concerning society. The study results demonstrate that 69.5% of the participants had any level of dog phobia, and the presence of dog phobia was at a statistically significant higher level in the 18–29 years age group and in those who did not own a pet, had never cared for stray animals, and had a personal or family history of having been attacked by a dog. It was stated by 53.3% of the participants that vaccinated and neutered dogs should be kept and cared for in shelters, and dog phobia was determined to be significantly higher in these individuals. There are articles in the literature related to the management of stray dogs. However, the distinguishing aspects of the current study are that the opinions of individuals were evaluated, dog phobia was studied in a nationwide sample, and the relationship between this and the management of stray dogs was determined.

In a previous study that evaluated attitudes toward stray dogs in three countries in Europe, it was reported that 53.7–90.6% of the participants had fed stray dogs (13). In the current study, 76.4% of the participants stated that they had previously cared for a stray animal (cat or dog), and the dog phobia rate was found to be significantly lower in those who had cared for stray animals. Another study that examined the source of the fear of dogs in children and adults showed that, in those who were previously exposed to dogs, there was a lower probability of developing long-term fear, even after a negative experience with dogs, and incident-free exposure to dogs at a young age could prevent fear from developing (14). The current study participants who owned a pet (cat or dog) were found to have significantly lower levels of dog phobia, and, as the duration of pet ownership increased, the levels of dog phobia decreased.

In research conducted in Europe, it was reported that 21.6% of participants in Bulgaria, 26.5% in Ukraine, and 4.2% in Italy had been attacked by a stray dog at some point in their life, and 9.2, 1.6, and 15.0%, respectively, stated that they or someone close to them had been bitten within the last year (13). Dog attacks can increase injury rates, especially in children (15). In the USA, dog bites are a significant source of injury in children, with approximately 914 new dog bite injuries requiring emergency department visits per day (16). In the current study, 46.1% of the participants reported that they or a family member had been attacked by a dog, and dog phobia was observed at a statistically significantly higher rate in those with a personal or family history of having been attacked by a dog. Dog bites not only cause physical injury but can also lead to long-term psychological problems. A previous study in Türkiye that evaluated injuries associated with dog bites reported that 65.8% of them were caused by stray dogs, and, in a psychiatric evaluation, post-traumatic stress disorder (PTSD) was found to have developed in 57.5% of the cases with injuries (17). A review examined the most common psychological outcomes of dog bites in children, and it was found that PTSD, dog phobia, anxiety, nightmares, and avoidance behaviors were the most common outcomes (18, 19).

In the current study, 67.8% of the participants stated that they were not afraid of owned dogs, 50.2% stated that they were afraid of stray dogs, 63.0% stated that they were afraid of being attacked by a stray dog, and 67.0% stated that they were afraid of contracting rabies from a stray dog. In a study that evaluated presentations at a rabies vaccination center in Türkiye between 2011 and 2019, 69% of the cases of contact with rabies risk were of dog origin, and 83.4% of the dogs were stray dogs (20). Injuries caused by dogs in New Zealand have been shown to have increased over time, and dog-related injuries have shown an increase close to 8-fold in a 40-year period (21). In Türkiye, the number of reported contacts with a risk of rabies was 186,466 in 2013, and this increased to 437,601 in 2023 (22). As contacts with the risk of rabies are mostly due to dogs (43.2%), this can be considered to be a factor in the high rate of dog phobia. A study in Europe reported that 10.8–40.9% of respondents stated that they “had felt threatened by dogs at some point,” 5.4–21.3% stated that they “feel physically threatened by free-roaming dogs,” and 14.8–36.3% stated that “free-roaming dogs are a threat to the safety of children.” University students in Algeria accepted that playing with an unknown dog was dangerous at a rate of 92.4, and 70.9% stated that they would not allow stray dogs to roam freely (23). In the evaluation of the dog phobia of the current study participants using the Specific Phobia Scale, any level of dog phobia (from mild–very severe) was observed with a rate of 69.5%. To the best of our knowledge, no previous study in the literature has evaluated the frequency of dog phobia. The finding that the rate of a personal or family history of having been attacked by a dog was higher than that in European countries could be associated with the rates of dog phobia being higher in Türkiye than in Europe. A previous study that investigated the sources of the fear of dogs found that, in adults with a fear of dogs, the fear had generally started in childhood, often with negative experiences with a dog as the initial cause of Pavlovian conditioning (e.g., having a painful or frightening experience with a dog), suggesting that conditioning alone is not the cause of fear (14).

The dog phobia status of the current study participants was compared according to sociodemographic data and descriptive characteristics related to animal ownership and care. No association was found between dog phobia and gender or education level, whereas a significant correlation was determined between geographic region distribution and dog phobia. It was found that dog phobia was more prevalent in the Eastern Anatolia Region (87.7%) compared to other regions, whereas it was at the lowest level in the Aegean (58.3%) and Mediterranean Regions (63.9%). This may be attributed to the fact that the Aegean and Mediterranean regions are neighboring the sea, have milder climates and pet ownership is more common in these regions. Especially in coastal regions, human-animal interaction occurs at an earlier age and more frequently, and positive experiences with dogs may have contributed to the decrease in phobia development. On the other hand, the fact that the Eastern Anatolia Region is far from the sea, has a colder climate and a mountainous geography, and that livestock breeding activities for economic purposes are carried out more in this region may lead to a more distant and cautious relationship with animals. In addition, the fact that stray animals, especially dogs, are perceived as more aggressive or herd animals acting with a protective instinct in the Eastern Anatolia Region, and that they are mostly raised in a task-oriented manner such as shepherd dogs may pave the way for individuals to develop a more fearful and cautious approach toward dogs. In this case, it can be considered as a possible environmental and cultural factor explaining the higher frequency of dog phobia in this region.

When the opinions of the participants about stray animal management were evaluated, it was found that 92.3% stated that all dogs should be vaccinated, 49.8% stated that all dogs should be neutered, 38.5% stated that neutered dogs should be returned to the streets, and 53.3% stated that vaccinated and neutered dogs should be held and cared for in shelters. A European study conducted in Bulgaria, Ukraine, and Italy evaluated attitudes toward stray dogs and dog ownership practices. The study results showed that the majority of participants wished to see fewer free-roaming dogs and thought that, for this to be successful, shelters should be provided, and the catch–neuter–release method and controlled breeding should be implemented (13). In a study of university students in Algeria, 94.1% stated that it is good to vaccinate dogs, 59.7% stated that it is not legal to vaccinate dogs against rabies, and 24.2% opposed neutering dogs to prevent rabies, but 59.2% stated that, when a dog had bitten a person, it should be captured and put down (23). Another study in Malaysia evaluated attitudes toward stray animal management; 35.7% of the participants emphasized that there should be neutering, and 33.7% stated that the trap–neuter–return method should be implemented (24).

The opinions of the participants on the management of stray animals with the presence of phobia and statistically significantly higher rates of dog phobia were observed in those who were undecided about whether all dogs should be neutered, those who stated that vaccinated and neutered dogs should not be left on the street again, and in those who stated that all vaccinated and neutered dogs should be kept and cared for in shelters. In a study conducted in Europe reported that young, male participants with no schooling or primary education wanted dogs to be neutered less often or more free- roaming dogs, whereas females defined as older or with a higher level of education, who felt threatened by free-roaming dogs, thought that it was necessary to prevent an increase in the numbers of stray dogs (13).

The right to life is a fundamental right for both animals and humans. In this context, in accordance with needs and societal resources, guidelines recommend the use of sustainable, humane, and effective methods in the management of the dog population while increasing the welfare of dogs and minimizing the adverse effects on the community (25). Legal dog control strategies can reduce the rates of dog bites (26). In a study in Australia that evaluated interventions to manage the dog population in 14 communities, significant increases in the rate of micro-chipped and neutered dogs were seen to be associated with significant decreases in dog attacks and the euthanasia of dogs in the participating communities over at least 3 years (27). There is a need for both time and new studies to be able to see the effect of the new practices in Türkiye on animals and dog phobia in society.

In our study, data were collected from across Türkiye through online access. The use of online access as the only data collection method in data collection may have affected the participation rate of the participants in online access due to reasons such as age, education level and internet access. Although the data were collected throughout the country, the rate of university graduate participants in our study was close to 90 per cent, and this distribution according to the current education level did not reflect the entire Turkish population, especially in terms of educational characteristics. For this reason, attention should be paid to the distribution of demographic characteristics in community-based studies. The data were collected by self-report method. This may result in effects such as recall bias. Participants may have reported their dog phobias or attitudes toward dog management differently than they actually did. Although a variety of demographic, experiential, and attitudinal factors were considered in our study, other psychological or environmental variables that may influence dog phobia (e.g., media exposure or personality traits) were not assessed. In this study, we examined the relationship between dog phobia and demographic and experiential factors using Multivariate Logistic Regression Analysis. In order to understand the relationships between phobia and attitudes, we included views about dog management in the model. However, the use of these variables as predictors of phobia presence may also be controversial. Dog phobia may shape an individual’s views on dog management and the inclusion of these variables in the model may contribute to understanding the relationship between phobia and attitudes. However, rather than assuming a direct causal relationship, our study aimed to investigate whether views on dog management are related to the presence of dog phobia. The inclusion of these variables in the model allows for a more comprehensive examination of potential behavioral and attitudinal patterns that may be linked to dog phobia. However, it should be recognized that attitudes may not only be influenced by phobia but also by cultural and individual experiences. Therefore, it would be useful for future research to examine these relationships in more detail with more comprehensive methodological approaches and different analysis techniques.

In conclusion, the results of this study show the following: dog phobia was observed at a high rate of 69.5%; dog phobia was significantly higher in younger participants, in those who had not previously cared for dogs, and in those with a negative experience with dogs; and 53.3% of the participants thought that vaccinated and neutered dogs should be kept and cared for in shelters, with dog phobia being significantly higher in these individuals. By showing the relationship between dog phobia frequency and demographic and experience factors, this study presents important data that will contribute to the better understanding of dog phobia in society and attitudes toward stray dogs. In this context, it should be kept in mind that dog phobia can be a determinant of attitude toward stray dogs, and there is a need for more detailed studies on this subject.

## Data Availability

The original contributions presented in the study are included in the article/Supplementary material, further inquiries can be directed to the corresponding author.

## Supplementary material

Supplementary Data Sheet 1 Data form.
