# Supplementary material for: The impact of dog phobia severity on views regarding stray dog management in Türkiye
Source: Front Vet Sci. 2025 May 7;12:1548580. doi: 10.3389/fvets.2025.1548580 (PMC12092426; doi:10.3389/fvets.2025.1548580)
Supplement: Supplementary Data Sheet 1 — Data form. [file Data_Sheet_1.pdf]

Please write the licence plate number of the province where you participated in the study (please specify with a number) …………….

What's your your Age? :……………..

What's your gender? **1)** Female **2)** Male

What's your education level? **1)** Primary school **2)** Middle school **3)** High School **4)** University and above

What's your marital status? **1)** Married **2)** Single (never married, widowed, divorced)

How many children do you have?...............

Do you own a pet? **1)** No **2)** Yes

If you have a pet, which animal do you have? (You can select more than one option)

1) Cat **2)** dog **3**) Other animals

How long have you had a pet? ……………./years

Have you ever cared for (fed, sheltered, etc.) a stray animal (cat-dog)? **1)** No **2)** Yes

Have you or a family member ever been attacked or bitten by a dog? **1)** No **2)** Yes

Please indicate your level of fear of dogs.

	**I am not afraid at all**	**I am not afraid**	**I am neither afraid nor unafraid**	**I am slightly afraid**	**I am very afraid**
Owned pet					
Stray dog					
The possibility of being attacked by a stray dog					
The possibility of contracting rabies from a stray dog					

What is your opinion on the management of stray animals (dogs)?

	**No**	**Undecided**	**Yes**
All dogs should be vaccinated			
All dogs should be neutered			
Dogs that have been vaccinated and neutered should be left on the streets again			
Dogs that have been vaccinated and neutered should be kept in shelters and given the necessary care			
